# “Non-invasive” brain stimulation is not non-invasive

**DOI:** 10.3389/fnsys.2013.00076

**Published:** 2013-12-23

**Authors:** Nick J. Davis, Martijn G. van Koningsbruggen

**Affiliations:** ^1^Department of Psychology, Swansea UniversitySwansea, UK; ^2^Centro Interdipartimentale Mente/Cervello, University of TrentoRovereto, Italy; ^3^Department of Cognitive Sciences, University of TrentoRovereto, Italy

**Keywords:** TMS, tDCS, non-invasive, neuroethics, safety, human

## Introduction

The functions of the healthy brain can be studied in two main ways. Firstly, the changes in the brain's state can be measured using techniques such as EEG or functional MRI. Secondly, the activity of the brain can be disrupted through the use of brain stimulation. The famous experiments of Wilder Penfield and colleagues in the 1950s showed the power of brain stimulation in people whose brain was exposed in surgery, and highlighted the possibility of inducing changes in the brain's state to demonstrate the involvement of specific brain areas in particular functions (Jasper and Penfield, 1954). Two main techniques are available for human brain stimulation: transcranial magnetic stimulation (TMS) and transcranial current stimulation (tCS). More recently, it has been suggested that TMS and tCS might be used to enhance brain function, as well as to disrupt activity.

These techniques have collectively become known as “non-invasive brain stimulation.” We argue that this term is inappropriate and perhaps oxymoronic, as it obscures both the possibility of side-effects from the stimulation, and the longer-term effects (both adverse and desirable) that may result from brain stimulation. We also argue that the established tendency for the effects of TMS and tCS to spread from the target brain area to neighboring areas is in itself contrary to the definition of non-invasiveness. We argue that the traditional definition of an invasive procedure, one which requires an incision or insertion in the body, should be re-examined, and we propose that it be widened to include targeted transcutaneous interventions.

## Types of brain stimulation

An electric current travelling through a coiled wire creates a magnetic field. This property is used in TMS to create brief magnetic pulses which easily traverse the skull and other matter overlaying the brain. The pulses generate electrical potentials in the brain, depolarizing neurons and thereby triggering action potentials (Di Lazzaro et al., 2004). A single pulse of TMS will have two effects: firstly the generation of action potentials in the targeted brain areas underlying the coil; secondly a refractory “silent period” in those same cells as the ion balance is restored. While the effects of a single TMS may only last on the order of a few milliseconds, multiple pulses may induce long term potentiation or depression in the target cells. For example, trains of pulses delivered at 1 Hz result in reduced excitability in the target area for a prolonged period of time, on the order of tens of minutes after the end of the stimulation. A recent development has been the use of rapid bursts of pulses such as theta-burst stimulation (TBS), which can have opposing effects on excitability depending on the temporal pattern of the bursts (Huang et al., 2005). TMS may be used either “online,” to affect the brain during a task, or “offline,” to compare task performance after vs. before longer periods of stimulation.

tCS is a term that covers several techniques, principally involving direct or alternating current (tDCS or tACS). In a typical tDCS experiment, the participant performs a task to establish a baseline performance level. Then a pair of electrodes is placed on the head, one (or both) of which overlie a target brain area. The experimenter delivers a small electrical current for around 10–20 min. Following this the participant performs the task a second time to establish whether the stimulation has had an effect on behavior. The effect depends on a number of factors, including current amplitude and duration (higher currents delivered for more time usually induce a greater effect), the polarity of the electrode over the target area (typically the negative electrode, or cathode, will worsen performance while the positive, anodal, electrode will enhance it), and the brain area and task under study (Nitsche and Paulus, 2000, 2001). tACS is less well studied, however the technique offers the possibility of exploring the casual involvement not only of a target brain area, but also of a particular frequency band. For example the beta range (15–35 Hz) is known to be associated with human motor control, however it has only recently been possible to show the causal involvement of beta frequencies in maintaining motor state (Pogosyan et al., 2009; Fuerra et al., 2011). These latter studies used tACS to increase the power of the beta band while the motor system was under study, giving somewhat contradictory results (Davis et al., 2012).

The timescale over which the effects of brain stimulation are seen can vary from milliseconds to weeks. At the briefest level, a single pulse of TMS lasts for 100–200 μs, during which time an electric field is induced in the target area. This is enough to generate action potentials in these target cells, and to induce a refractory silent period following the initial burst. Conversely the instantaneous effects of tCS are under-explored, and much of our knowledge of the effects of the electric field on the brain comes from modeling studies (e.g., Miranda et al., 2006). Most experimental uses of brain stimulation involve medium-scale effects, which occur on the order of minutes to hours. tDCS experiments exploit the polarizing effect of the electric field on the resting membrane potential, which lasts for around 90 min following 13 min of stimulation (Nitsche and Paulus, 2001). Similarly, the effects of TBS may last up to 1 h (Huang et al., 2005), which can be extended to more than 2 h by slightly adjusting the TBS protocol (Nyffeler et al., 2006).

There is considerable interest in the therapeutic possibilities of brain stimulation. Both TMS and tDCS have shown some success in the treatment of depression (Slotema et al., 2010), stroke (Hummel and Cohen, 2006), and tinnitus (Fregni et al., 2006). Most usefully for clinical applications, certain regimes of brain stimulation may lead to longer-lasting changes in brain function. A particularly effective strategy for generating lasting effects is to apply stimulation in multiple sessions spaced around 24 h apart. This regime makes brain stimulation a possible adjunct therapy for neurological disorders, which can be administered in outpatient clinics, leaving the patient free to return home between stimulation sessions.

## Safety issues in brain stimulation

No brain stimulation technique is completely free of side-effects. The complications of surgical procedures such as deep brain stimulation (DBS) are well-monitored and well-understood in the context of weighing the potential benefit to the patient, and are considered in terms of short-term and longer-term effects (Beric et al., 2002). While the safety limits for brain stimulation are reasonably well mapped (Nitsche et al., 2003; Bikson et al., 2009) there remain real risks of seizure from TMS and tCS, and scalp burns from tCS, if appropriate care is not taken. However, the greater risk comes from the “known unknowns” of brain stimulation: unplanned effects from build-up of stimulating effects in non-target areas, or from build-up of effects across multiple sessions. This latter risk can also be an advantage, as discussed above, however inducing long-lasting changes in cortical excitability can be dangerous to the participant if not properly controlled. Indeed, many institutions that use brain stimulation insist on a minimum interval between sessions to prevent a build-up of effects.

Brain stimulation of healthy volunteers has recently become an established tool for the study of diverse features of the human brain, from basic neurophysiology (Stagg et al., 2009) to large-scale networks (Polanía et al., 2011). It is clear that brain stimulation is a powerful tool in the hands of neuroscientists, and the use and utility of these techniques will increase as we learn more about their effects on the brain and about the optimum parameters for generating these effects. A key feature of brain stimulation is that the effects of stimulation can greatly outlast the stimulation phase, sometimes up to several weeks after the end of a stimulation session (e.g., Boggio et al., 2007).

## Common definitions of invasiveness

In opposing the term “non-invasive,” we must examine the definition of the term. The common, intuitive definition of the term “non-invasive” implies a procedure where no incision or insertion is made into the body. In most medical contexts this is a sensible distinction; physicians must balance the risks and benefits of invasive and non-invasive procedures for both monitoring (e.g., Shoemaker et al., 1998) and treating (e.g., Medoff, 2008) medical complaints. However, invasiveness is not restricted to this definition alone. For example, the *Oxford English Dictionary* gives two relevant usages for the term “non-invasive”: “*Chiefly Med[ical] Esp[ecially] of a neoplasm or microorganism: not spreading into adjacent tissue from an initial site of development or colonization. Also: designating or relating to such a pattern of growth*”; and “*Med[ical] Of a diagnostic or therapeutic procedure: that does not require the insertion of instruments (often including hypodermic needles) through the skin or into a body cavity”* (“non-invasive,” Oxford English Dictionary, 3rd Edition, 2003).

The second of these dictionary definitions is what we think of as the intuitive neuroscientific definition of “non-invasiveness.” Clearly, in contradistinction to surgical procedures such as DBS or direct cortical stimulation, the application of a coil or electrode to the scalp is non-invasive in the sense that the instrument does not physically enter the body. We believe that transcranial brain stimulation with TMS or tCS fits better with the former definition. Induced currents spread from the point of delivery through the brain (and nearby tissues) to adjacent regions. This spread is large in the case of tCS (Miranda et al., 2006), compared to a relatively focal sphere of stimulation in the clearly invasive procedure of DBS (Butson et al., 2006). This unintended and unwanted current spread is consistent with the dictionary definition's sense of diffusion away from a source region. We would similarly classify novel techniques such as optogenetics as not being non-invasive, since the stimulation (light) must pass through multiple layers of tissue, and possibly beyond, to activate the target cells (leaving aside the problem of introducing photosensitive proteins into the tissue: Fenno et al., 2011).

We do not suggest that invasiveness in its own right should preclude researchers from using a technique; as we have seen, TMS and tCS are safe when used correctly. Referring to brain stimulation techniques as “non-invasive” likely increases the palatability of the techniques to non-expert participants; an important factor in recruitment, as “brain stimulation” already somewhat raises the stakes when recruiting for experiments or trials. We do not suggest that recruitment adverts should advertise “invasive brain stimulation,” rather that the use of the term “non-invasive” may create an illusion of comfort in participants' and non-experts' minds that may not be warranted. We therefore propose that TMS and tCS be referred to simply as “brain stimulation,” without the potentially misleading qualifier of “non-invasive.”

## Widening access to brain stimulation

The widening use of brain stimulation has been much discussed recently. In particular, the relatively low cost and ease of manufacture of tDCS has led to something of a movement in so-called “DIY-tDCS” for self-stimulation. Electrical brain stimulation has been suggested as a promising option for improving human experience in a number of domains, including: numerical skills (Cohen Kadosh et al., 2010; Snowball et al., 2013); sport (Banissy and Muggleton, 2013; Davis, 2013); memory capacity (Hoy et al., 2013); and depression (Nitsche et al., 2009). As positive results trickle out of labs and clinics, the likelihood is that a greater number of people will wish to explore the use of brain stimulation. As technologies improve and become more widespread, the ethical implications of (mis)use of brain stimulation must be considered; this concern has not been thoroughly addressed in relation to brain stimulation (but see Green et al., 1997; Cohen Kadosh et al., 2012).

Recent works have attempted to address the ethical and policy implications of widespread use of enhancing technologies. For example, Fitz and Reiner (2013) propose a stance of “managed technological optimism,” whereby stakeholders (including DIY-tDCS developers) take a share in responsibly determining guidelines for using neuroenhancing technology. We see the issue of self-treatment for neurally-mediated disorders as a potentially more serious issue, as people who are not satisfied with physician-delivered treatment seek adjunct treatment with brain stimulation, without clear guidance about proper controls or interactions with existing treatments (Cabrera et al., 2013; Davis et al., 2013). Potential users should be aware that guidelines and principles have been published that address the safe use of brain stimulation techniques (e.g., Green et al., 1997; Rossi et al., 2009; Davis et al., 2013).

## Conclusions

Brain stimulation will continue to develop, to the benefit of scientists and of patients, and we foresee its routine use in clinics. We propose that the term “non-invasive brain stimulation” no longer be used, as the term may mislead non-expert users into the view that the effect of the technique is necessarily mild. Any technique which directly affects brain tissue to generate such powerful acute and long-lasting effects should be treated with the same respect as any surgical technique, and proper safety and ethical guidelines should apply in institutions where brain stimulation is in use. We would draw an analogy between brain stimulation and gamma-knife radiotherapy, which is also “non-invasive” in the sense that no incisions or insertions are made in the person, but clinicians and the public have a healthy and proper respect for the nature of the technique. We propose also that researchers take care to develop good and safe practice for the use of their research, and be mindful that in a climate of wide and open dissemination of scientific results, exciting, and beneficial results will reach well beyond the labs and clinics.
